# Supplementary material for: RIF1 integrates DNA repair and transcriptional requirements during the establishment of humoral immune responses
Source: Nat Commun. 2025 Jan 17;16:777. doi: 10.1038/s41467-025-56166-5 (PMC11742068; doi:10.1038/s41467-025-56166-5)
Supplement: Supplementary file 1 — Supplementary Information [file 41467_2025_56166_MOESM1_ESM.pdf]

Supplementary Information

RIF1 integrates DNA repair and transcriptional requirements during the establishment of humoral immune responses

Eleni Kabrani[1#], Ali Rahjouei[1,6#], Maria Berruezo-Llacuna[1,2], Svenja Ebeling[1,3], Tannishtha Saha[1,3], Robert Altwasser[1,7], Veronica Delgado-Benito[1], Rushad Pavri[4], and Michela Di Virgilio[1,5,*]

[#]These authors contributed equally

*Correspondence: michela.divirgilio@mdc-berlin.de

This PDF file includes:

Supplementary Fig. 1-6

Supplementary Table 1

Supplementary References

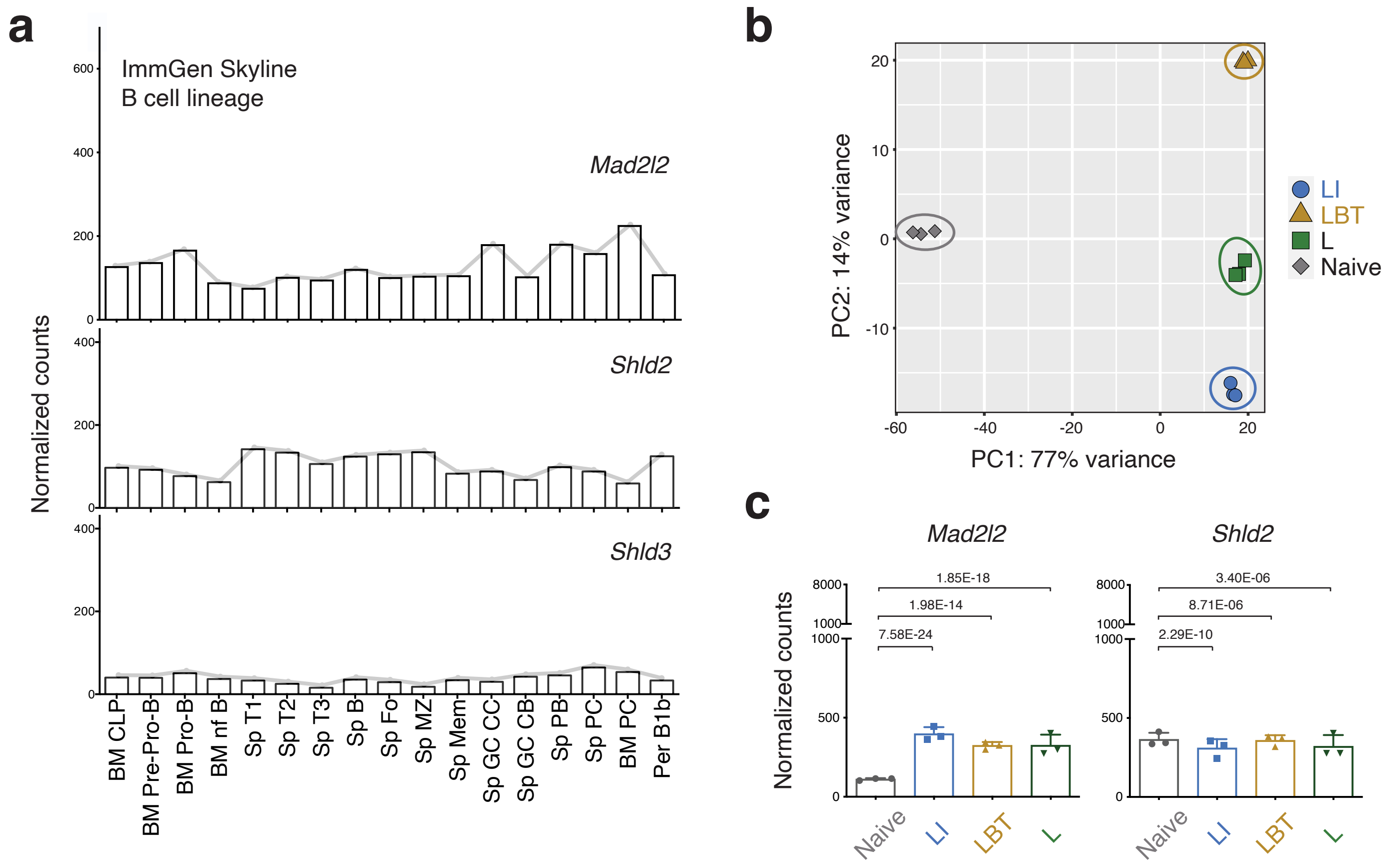

Supplementary Figure 1. Stimulation-dependent clustering of B cell transcriptomics profiles after *ex vivo* activation. a Expression of *Mad2l2*, *Shld2* and *Shld3* genes across B cell lineage developmental stages as determined by the Immunological Genome Project (ImmGen) Skyline RNA-Seq analysis. BM: bone marrow; CLP: common lymphoid progenitor; nf: newly-formed; Sp: splenic; Fo: follicular; MZ: marginal zone; Mem: memory; GC: germinal center; CC: centrocytes; CB: centroblasts; PB: plasmablasts; PC: plasma cells; Per B1b: peritoneal B1b. **b** Principal component analysis for the RNA-Seq datasets of WT splenocytes before (naive) and after 48 h stimulation with LPS and IL-4 (LI), LPS, BAFF and TGFβ (LBT), or LPS only (L). The RNA-Seq analysis was performed on three biological replicates/mice per condition. **c** Expression of *Mad2l2* and *Shld2* in naive and LI/LBT/L-stimulated B cells. *Shld3* expression levels were below detection in all datasets. Expression values in panels **a** and **c** were normalized by DESeq2. The adjusted p-value of significant differences between samples in panel **c** is indicated. Source data are provided as a Source Data file. Related to Figure 1.

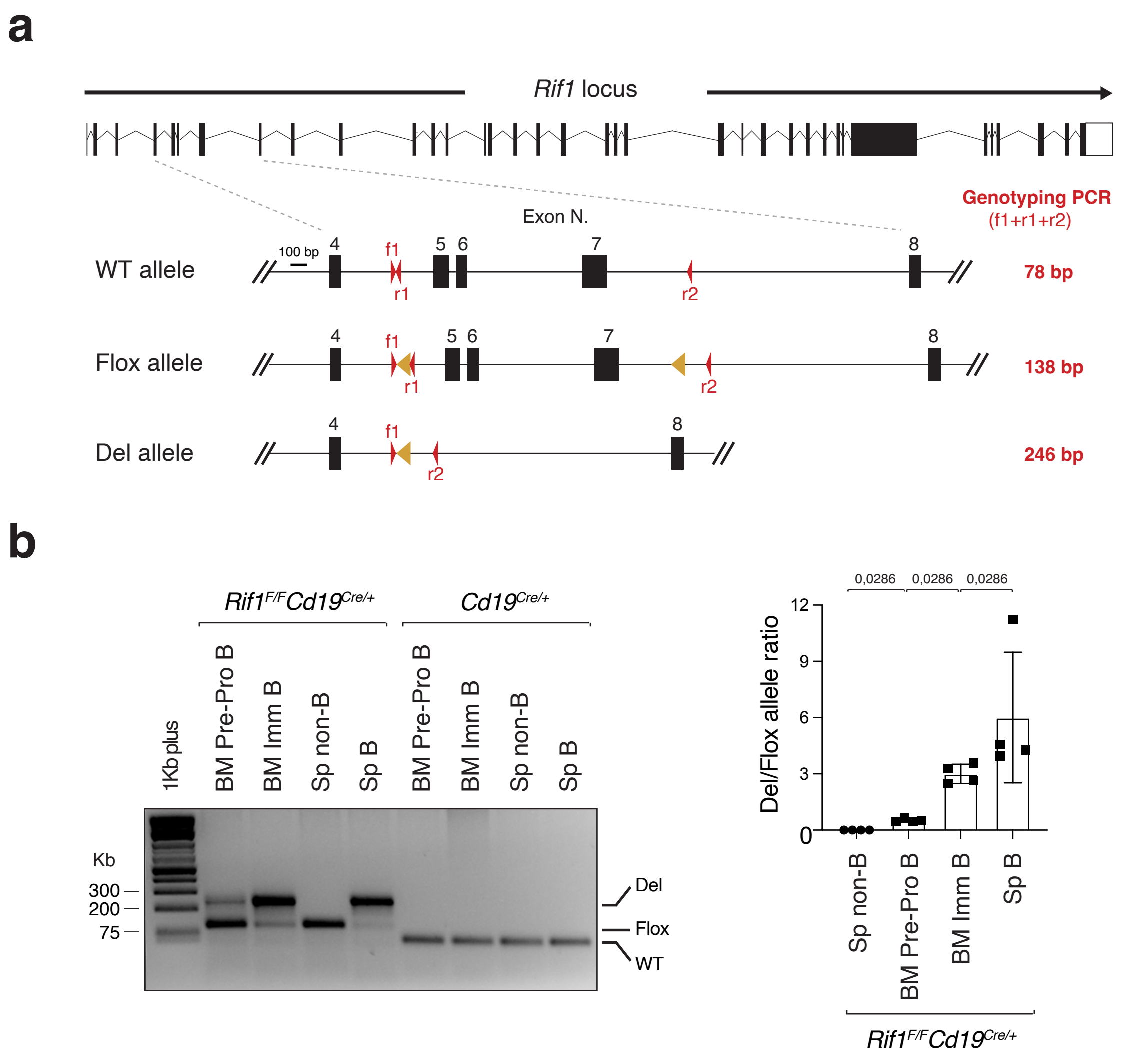

Supplementary Figure 2. *Rif1$^{F/F}$Cd19$^{Cre/+}$* mice conditionally delete *Rif1* in the B cell lineage by the immature cell stage. **a** Schematic representation of the mouse *Rif1* locus (Ensembl Rif1-201 ENSMUST00000112693.10) in the WT, conditional (Flox) and deleted (Del) null allele configurations, with the LoxP sites shown as yellow triangles. Genotyping PCR primers and expected bands are indicated in red. Adapted from Buonomo et al. 2009. **b** Left: PCR analysis of the *Rif1* allele status in bone marrow (BM) Pre-Pro B cells, BM immature B cells (Imm B), splenic (Sp) non-B cells and Sp B cells, sorted from mice of the indicated genotype. Right: Summary graph showing semi-quantitative assessment of floxed *Rif1* allele's deletion in four *Rif1$^{F/F}$Cd19$^{Cre/+}$* mice displayed as ratio between the Del and Flox bands' intensities. Significance in panel **b** was calculated with the Mann–Whitney U test with error bars representing SD. * = $p \leq 0.05$. Source data are provided as a Source Data file. Related to Figure 2.

Supplementary Figure 3

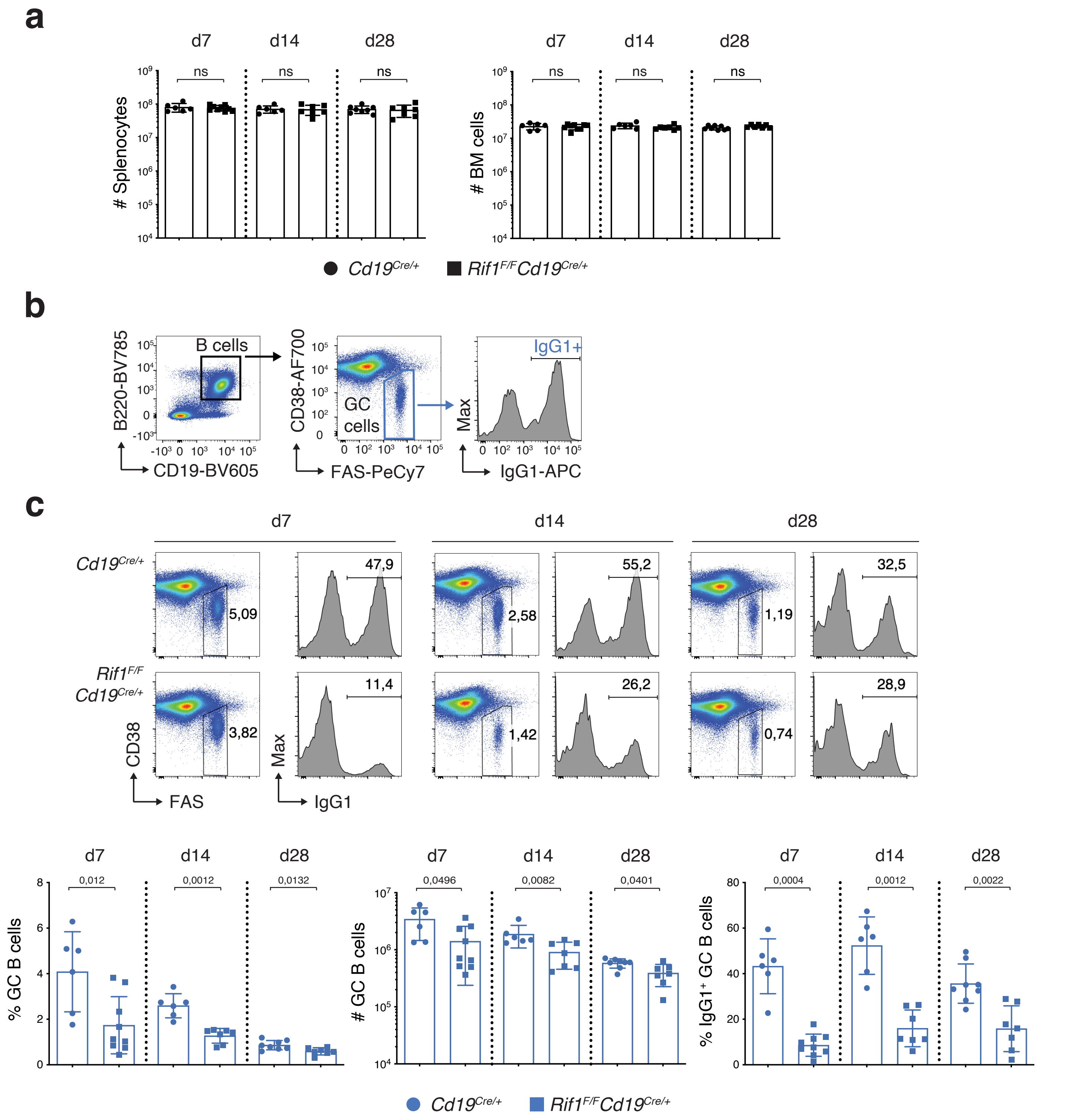

Supplementary Figure 3. RIF1 deficiency results in a reduced number of class-switched GC B cells upon immunization. a Total spleen and bone marrow cellularity of the *Cd19*$^{Cre/+}$ and *Rif1*$^{F/F}$*Cd19*$^{Cre/+}$ mice in the NP-CGG immunization groups from Figure 4. NP-CGG: 4-hydroxy-3-nitrophenylacetyl hapten conjugated to Chicken Gamma Globulin; BM: bone marrow. **b** Gating strategy for Germinal Center (GC) analysis. **c** Top: Representative flow cytometry plots measuring the percentage of GC cells and IgG1-class switched GC cells in spleens of *Cd19*$^{Cre/+}$ and *Rif1*$^{F/F}$*Cd19*$^{Cre/+}$ mice at the indicated days after immunization. Bottom: Summary graphs for n = nine *Rif1*$^{F/F}$*Cd19*$^{Cre/+}$ mice at d7, eight *Cd19*$^{Cre/+}$ at d28, seven *Rif1*$^{F/F}$*Cd19*$^{Cre/+}$ at d14 and d28, six *Cd19*$^{Cre/+}$ at d7 and six *Cd19*$^{Cre/+}$ at d14 in at least three independent experiments. Significance in panels **a** and **c** was calculated with two-sided Mann–Whitney U test and error bars represent SD. ns: not significant. Source data are provided as a Source Data file. Related to Figure 4.

Supplementary Figure 4

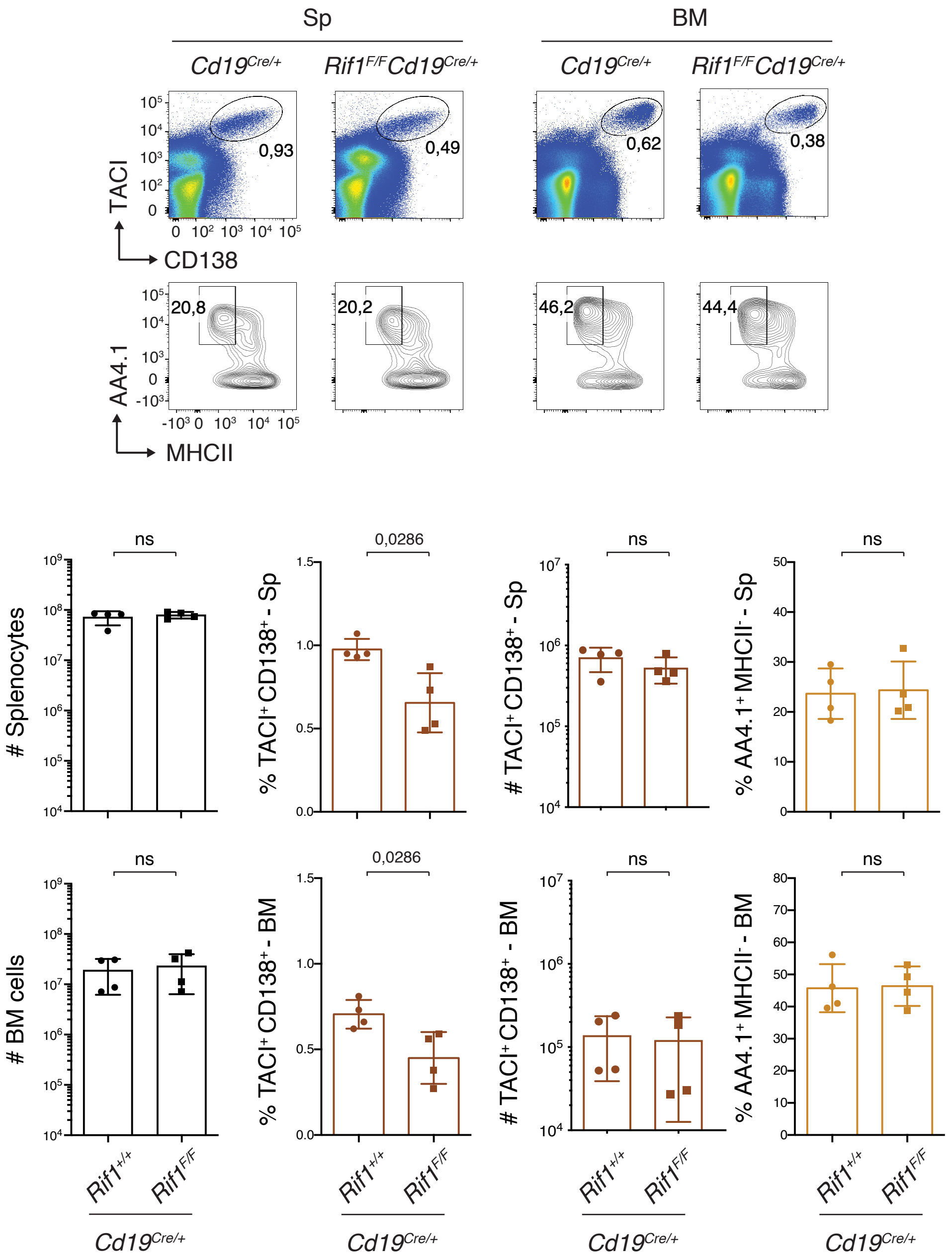

Supplementary Figure 4. RIF1 ablation does not result in detectable changes of the plasma cell compartment in steady state condition. Top: Representative flow cytometry plots measuring percentage of antibody secreting cells and plasma cells in spleens and bone marrows of unimmunized mice of the indicated genotypes. The same gating strategy as in Figure 4a was employed. Sp: spleen; BM: bone marrow. Bottom: Summary graphs for four mice per genotype. Significance was calculated with two-sided Mann–Whitney U test, and error bars represent SD. ns: not significant. Source data are provided as a Source Data file. Related to Figure 4.

Supplementary Figure 5

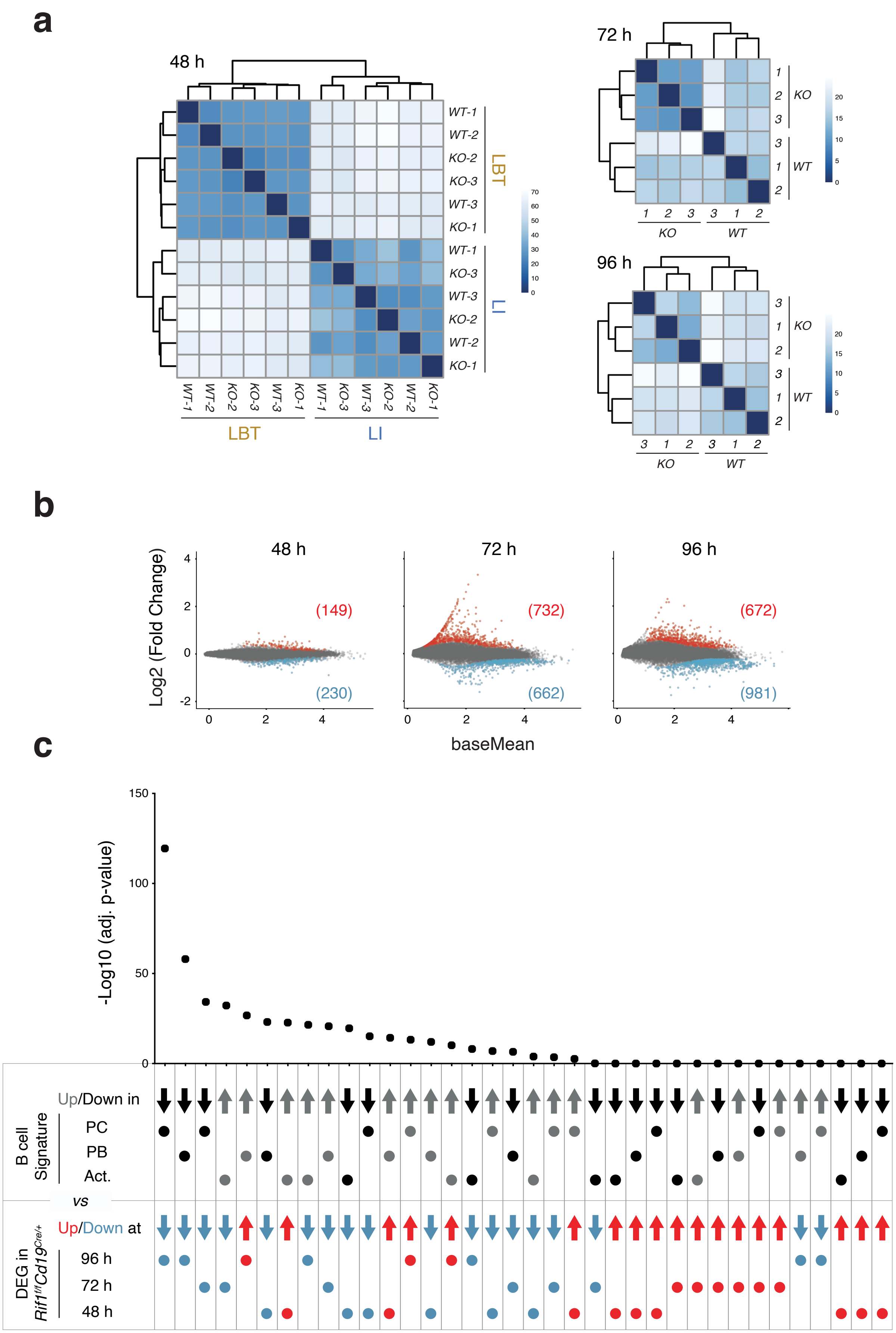

Supplementary Figure 5. RIF1 deficiency alters the transcriptional landscape of activated B cells. a-b Dendrograms (**a**) and scatterplots (**b**) for the RNA-Seq datasets generated according to the experimental scheme depicted in Fig. 5a. For the 48 h time point, the analysis was performed on both LI- and LBT-stimulated cultures. Data summarizes results from three mice per genotype per stimulation condition. In the scatterplots, genes with an adjusted p-value <= 0.05 that are up- or down-regulated in *Rif1*$^{F/F}$-*Cd19*$^{Cre/+}$ cells are highlighted in red or blue, respectively, and the number of DEGs per category is included in parenthesis. **c** Statistical significance of pairwise comparisons between the indicated gene lists *via* hypergeometric analysis. Related to Figure 5.

Supplementary Figure 6

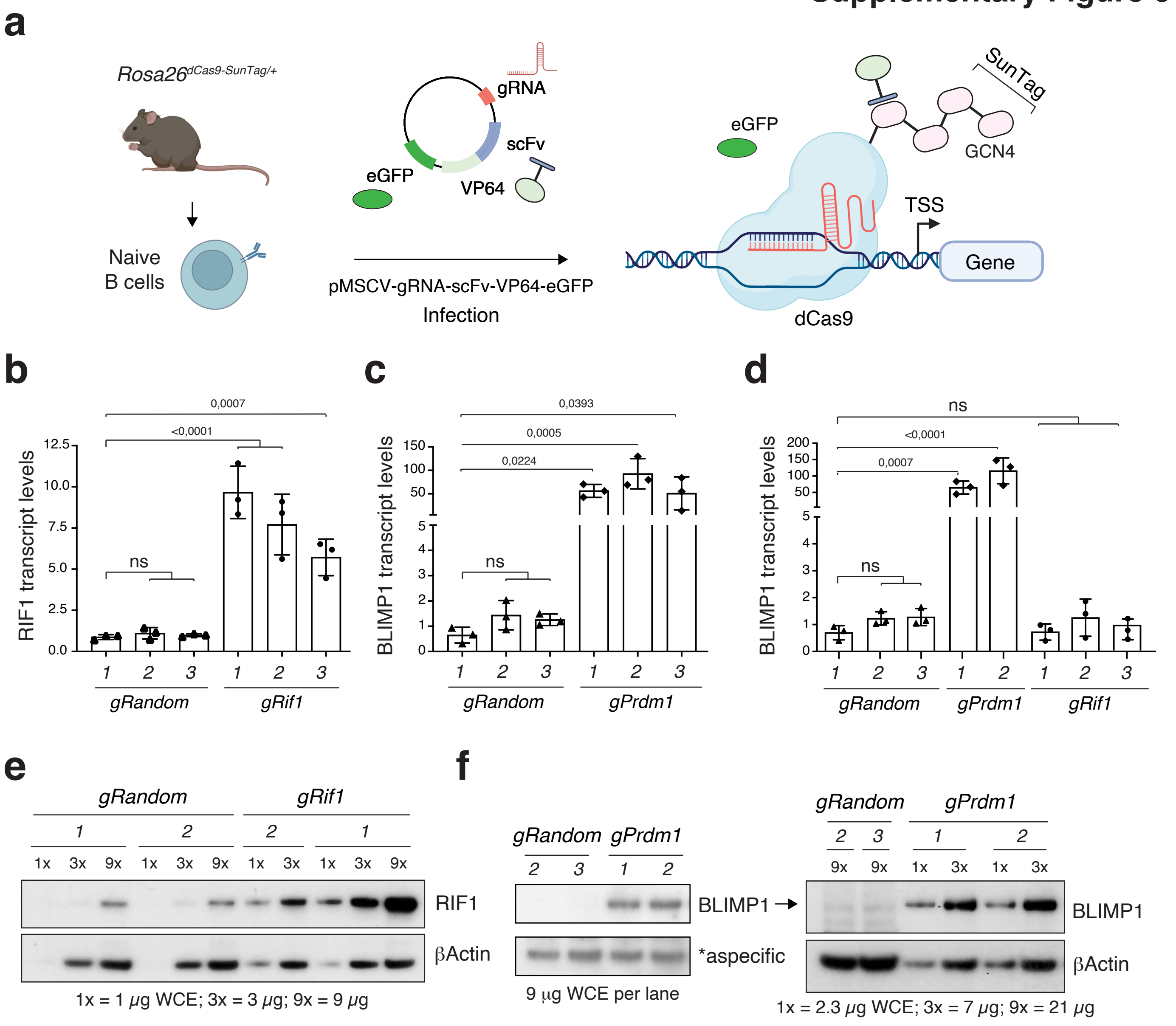

Supplementary Figure 6. CRISPRa-mediated overexpression of RIF1 does not alter *Prdm1* expression in *ex vivo* activated B cells. a Schematic representation of the CRISPRa experimental setup in primary B cell cultures. *Rosa26*$^{dCas9\text{-}SunTag/+}$ mice constitutively express the nuclease-deficient Cas9 (dCas9) fused to a stretch of ten GCN4 peptides (SunTag). To achieve target gene upregulation, *Rosa26*$^{dCas9\text{-}SunTag/+}$ B cells were transduced with the construct coding for scFv (single-chain variable fragment), which recognises GCN4, fused to the transcriptional activator VP64. TSS: transcription start site. **b-d** qPCR analysis of *Rif1* (b) and *Prdm1* transcripts (c-d) in sorted *Rosa26*$^{dCas9\text{-}SunTag/+}$ GFP$^+$ B cells. The graphs summarize the results from three independent experiments with each dot representing a biological replicate generated by pooling cells isolated from two mice. Values are expressed as fold change over the mean expression of all gRandom controls, which was set to 1. **e-f** Western blot analyses of whole cell extracts (WCE) from sorted *Rosa26*$^{dCas9\text{-}SunTag/+}$ GFP$^+$ B cells. The blots are representative of three gRNAs employed per condition and three independent experiments (every biological replicate is a pooled sample from two mice as in b-d). The amounts of WCE used per lane are specified below each panel. The arrow between the two anti-BLIMP1 blots in panel f indicates that samples have been loaded again in different WCE amounts to better highlight BLIMP1 protein levels across conditions. Significance in panels **b**, **c**, and **d** was calculated with one-way Anova test (multiple comparisons) and error bars represent SD. ns: not significant. Source data are provided as a Source Data file. Created in BioRender. Di virgilio, M. (2025) BioRender.com/f60c337. Related to Figure 8.

RIF1 and BLIMP1 expression analysis by Western Blot (uncropped - Related to Suppl.Fig.6)

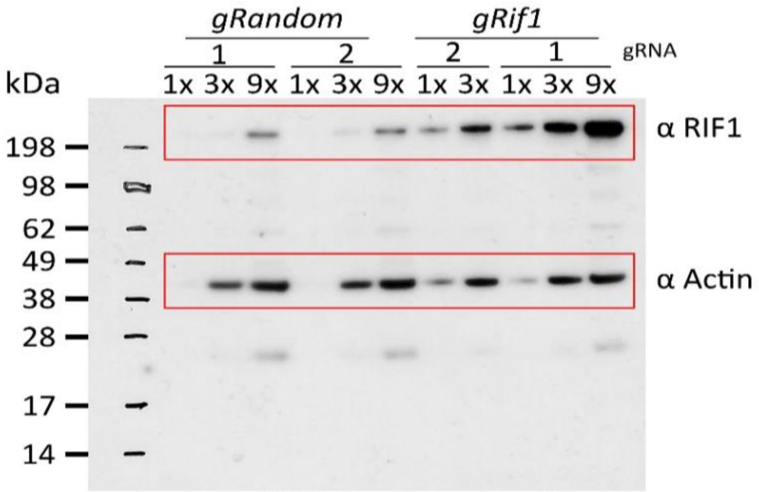

1x = 1 µg WCE; 3x = 3 µg; 9x = 9 µg
α RI1 & α Actin

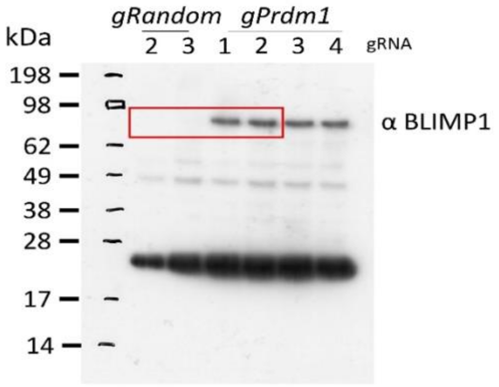

9 µg WCE, α BLIMP1

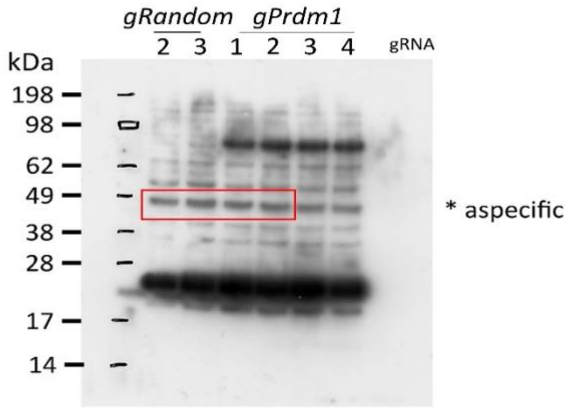

9 µg WCE, α BLIMP1

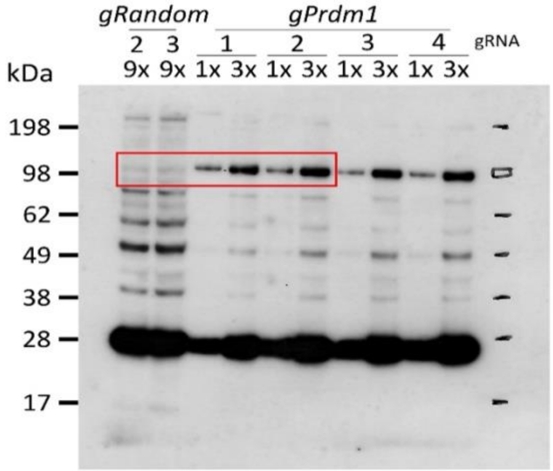

1x = 2.3 µg WCE; 3x = 7 µg; 9x = 21 µg
α BLIMP1

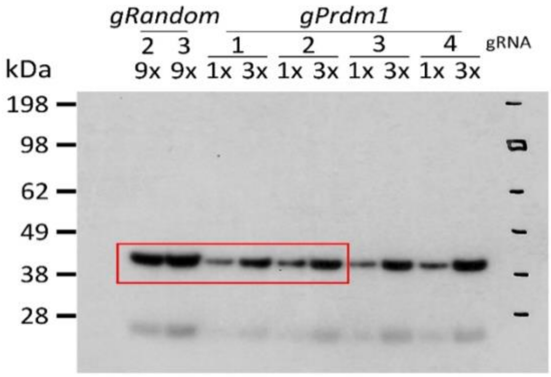

1x = 2.3 µg WCE; 3x = 7 µg; 9x = 21 µg
α Actin

Abbreviations: kDa: kilodalton, WCE: whole cell extract

Supplementary Table 1. List of oligonucleotides used in this study.

Genotyping PCR

Primers	**Sequence (5'→3')**	**Reference**
Rif1-f1	TTAGAGGAACTGAGGGGTAGGTAG	Buonomo et al, JCB 2009
Rif1-r1	AACTGCAACTCTACTGAGGGAAG	Buonomo et al, JCB 2009
Rif1-r2	TGAAACCGTAGCCAGAAACTG	Buonomo et al, JCB 2009

ChIP-qPCR

Primers	**Sequence (5'→3')**	**Region**	**Reference**
MDV_p1372 (Fw)	ATTGTTGGACACTGAGAGGAGG	peak_5084 (*Ccr7*)	This paper
MDV_p1373 (Rev)	TGGCACGAGGTTTAGATGAGTA	peak_5084 (*Ccr7*)	This paper
MDV_p1374 (Fw)	AGTACCAACTCGCTTGTGAAAG	peak_15625 (*Cd44*)	This paper
MDV_p1375 (Rev)	TTGTGGCGCCTTCTCATAAAAG	peak_15625 (*Cd44*)	This paper
MDV_p1376 (Fw)	GAAACCCGCCTCATTTTCAGTA	peak_17595 (*Fcgr1*)	This paper
MDV_p1377 (Rev)	AGAAGATTCAAAGTCCCGCCTA	peak_17595 (*Fcgr1*)	This paper
MDV_p1430 (Fw)	TATCTACTTCCGCTTCCGCTAC	peak_18712 (*Exosc3*)	This paper
MDV_p1431 (Rev)	ATGGGATGAAGCGAGTATCTCC	peak_18712 (*Exosc3*)	This paper
MDV_p1432 (Fw)	TCACTCTGCAACTCATAGCTTTG	Negative region downstream peak_18712 (*Exosc3*)	This paper
MDV_p1433 (Rev)	TTCAGAGGTTTAGTGTGACAGGA	Negative region downstream peak_18712 (*Exosc3*)	This paper
MDV_p1378 (Fw)	CGTGGTCTAAGGGCATTTGT	Negative region downstream peak_17595 (*Fcgr1*)	This paper
MDV_p1379 (Rev)	CCCCAAACTCCCACACTCTA	Negative region downstream peak_17595 (*Fcgr1*)	This paper
MDV_p1306 (Fw)	AGTTCAACCTCACAGACACTCT	peak_24072 (*Cd37*)	This paper
MDV_p1307 (Rev)	AAAGAGAGAGACCCAGGGAAG	peak_24072 (*Cd37*)	This paper
MDV_p1316 (Fw)	TGATTGGCCTCGTCTCTTAACT	peak_18460 (*Cdh17*)	This paper
MDV_p1317 (Rev)	AGTCCACCATAGCCTGAAAGTC	peak_18460 (*Cdh17*)	This paper

MDV_p1328 (Fw)	AGTACTGGAGATGACAAGGTCC	peak_25817 (*Klf2*)	This paper
MDV_p1329 (Rev)	CTGCACCCCAAAGTCTAACAAC	peak_25817 (*Klf2*)	This paper
MDV_p1336 (Fw)	CCAGAGTCGAATATCTACGCCT	peak_27144 (*Tle3*)	This paper
MDV_p1337 (Rev)	GAGATGGTGAAGAAGGGAAAGC	peak_27144 (*Tle3*)	This paper

CRISPRa

gRNA	**Sequence (5’→3’)**	**Reference**
gRif1_1	CACCGGGCCCCCGGAGCACCTCGGG	Horlbeck et al, eLife 2016
gRif1_2	CACCGGGGCGCGCCGGGCTGCTCGA	Horlbeck et al, eLife 2016
gRif1_3	CACCGGCCAACGGGCGGTGGCGCGC	Horlbeck et al, eLife 2016
gPrdm1_1	CACCGGGCCGCGGGTCGCAGTCGGT	Horlbeck et al, eLife 2016
gPrdm1_2	CACCGGGCGGGTCGCAGTCGGTGGG	Horlbeck et al, eLife 2016
gPrdm1_3	CACCGGCAAACAGAGGAAGCTGCCG	Horlbeck et al, eLife 2016
gRandom_1	CACCGGCTTTCACGGAGGTTCGACG	Horlbeck et al, eLife 2016
gRandom_2	CACCGATGTTGCAGTTCGGCTCGAT	Horlbeck et al, eLife 2016
gRandom_3	CACCGACGTGTAAGGCGAACGCCTT	Horlbeck et al, eLife 2016

qPCR

Primers	**Sequence (5’→3’)**	**Reference**
Rif1 (Fw)	CACCGGGCCCCCGGAGCACCTCGGG	Li et al, Cell Regen 2022
Rif1 (Rev)	CACCGGGGCGCGCCGGGCTGCTCGA	Li et al, Cell Regen 2022
Prdm1 (Fw)	CACCGGCCAACGGGCGGTGGCGCGC	Xiao et al, Int. J. Mol. Med. 2019
Prdm1 (Rev)	CACCGGGCCGCGGGTCGCAGTCGGT	Xiao et al, Int. J. Mol. Med. 2019
Ubc (Fw)	GCCCAGTGTTACCACCAAGA	Albershardt et al, J Immunol methods, 2012
Ubc (Rev)	CCCATCACACCCAAGAACA	Albershardt et al, J Immunol methods, 2012

Supplementary References

1. Albershardt, T. C., Iritani, B. M. & Ruddell, A. Evaluation of reference genes for quantitative PCR analysis of mouse lymphocytes. *J. Immunol. Methods* **384**, 196–199 (2012).
2. Xiao, J. *et al.* Downregulation of Blimp1 inhibits the maturation of bone marrow-derived dendritic cells. *Int. J. Mol. Med.* **43**, 1094–1104 (2019).
3. Li, L. *et al.* Rif1 interacts with non-canonical polycomb repressive complex PRC1.6 to regulate mouse embryonic stem cells fate potential. *Cell Regen.* **11**, 25 (2022).
4. Horlbeck, M. A. *et al.* Compact and highly active next-generation libraries for CRISPR-mediated gene repression and activation. *eLife* **5**, e19760 (2016).
5. Buonomo, S. B. C., Wu, Y., Ferguson, D. & de Lange, T. Mammalian Rif1 contributes to replication stress survival and homology-directed repair. *J. Cell Biol.* **187**, 385–398 (2009).
