# Supplementary material for: RIF1 integrates DNA repair and transcriptional requirements during the establishment of humoral immune responses
Source: Nat Commun. 2025 Jan 17;16:777. doi: 10.1038/s41467-025-56166-5 (PMC11742068; doi:10.1038/s41467-025-56166-5)
Supplement: Supplementary file 2 — Description of Additional Supplementary Files [file 41467_2025_56166_MOESM2_ESM.pdf]

Description of Additional Supplementary Files

Supplementary Data 1

Pairwise comparison of differentially regulated genes in control (*Cd19*$^{Cre/+}$) *versus* *Rif1*$^{F/F}$*Cd19*$^{Cre/+}$ B lymphocytes. Related to Figures 5 and S5.

Supplementary Data 2

Lists of genes in the PB and PC transcriptional signatures that are differentially regulated in *Rif1*$^{F/F}$*Cd19*$^{Cre/+}$ activated B cells. Related to Figure 5.

Supplementary Data 3

List of RIF1 peaks in the genome of *ex vivo* activated B cells as determined by ChIP-Seq analysis. Related to Figure 6.

Supplementary Data 4

Lists of RIF1-bound genes in the top GREAT gene ontology enrichment categories. Related to Figure 6.

Supplementary Data 5

List of potential RIF1 binding motifs identified by the *de novo* motif–discovery program MEME-ChIP. Related to Figure 6.

Supplementary Data 6

List of motifs identified in RIF1 and BLIMP1 co-occupied regions by the motif–enrichment program SEA. Related to Figure 7.

Supplementary Data 7

List of RIF1 and BLIMP1 target genes. Related to Figure 8.
