# Supplementary material for: RIF1 integrates DNA repair and transcriptional requirements during the establishment of humoral immune responses
Source: Nat Commun. 2025 Jan 17;16:777. doi: 10.1038/s41467-025-56166-5 (PMC11742068; doi:10.1038/s41467-025-56166-5)
Supplement: Supplementary file 10 — Reporting Summary [file 41467_2025_56166_MOESM10_ESM.pdf]

nature portfolio

Corresponding author(s): Michela Di Virgilio

Last updated by author(s): Nov 8, 2024

Reporting Summary

Nature Portfolio wishes to improve the reproducibility of the work that we publish. This form provides structure for consistency and transparency in reporting. For further information on Nature Portfolio policies, see our Editorial Policies and the Editorial Policy Checklist.

Statistics

For all statistical analyses, confirm that the following items are present in the figure legend, table legend, main text, or Methods section.

n/a	Confirmed	
☐	☒	The exact sample size (*n*) for each experimental group/condition, given as a discrete number and unit of measurement
☐	☒	A statement on whether measurements were taken from distinct samples or whether the same sample was measured repeatedly
☐	☒	The statistical test(s) used AND whether they are one- or two-sided<br>*Only common tests should be described solely by name; describe more complex techniques in the Methods section.*
☒	☐	A description of all covariates tested
☒	☐	A description of any assumptions or corrections, such as tests of normality and adjustment for multiple comparisons
☐	☒	A full description of the statistical parameters including central tendency (e.g. means) or other basic estimates (e.g. regression coefficient) AND variation (e.g. standard deviation) or associated estimates of uncertainty (e.g. confidence intervals)
☐	☒	For null hypothesis testing, the test statistic (e.g. *F*, *t*, *r*) with confidence intervals, effect sizes, degrees of freedom and *P* value noted<br>*Give P values as exact values whenever suitable.*
☒	☐	For Bayesian analysis, information on the choice of priors and Markov chain Monte Carlo settings
☒	☐	For hierarchical and complex designs, identification of the appropriate level for tests and full reporting of outcomes
☐	☒	Estimates of effect sizes (e.g. Cohen's *d*, Pearson's *r*), indicating how they were calculated

Our web collection on statistics for biologists contains articles on many of the points above.

Software and code

Policy information about availability of computer code

Data collection	NGS data was acquired using Illumina sequcing platforms or was download from public repositories. Accessions are provided in the "Data availability" section.

Data analysis	RNA-seq: Sequencing data quality confirmed by generating quality control report using FastQC(Galaxy Version 0.74). For Quality and adapter trimming Trim Galore (Galaxy version 0.6.7) was used. RNA STAR, a Gapped-read mapper for RNA-seq data(Galaxy Version 2.7.11a) was used. featureCounts (Galaxy Version 2.0) was used to measure gene expression in RNA-Seq experiments from BAM files. DESeq2 (Galaxy Version 2.11.40.8) was used to determines differentially expressed features from count tables.<br><br>ChIP-seq as described below: Sequencing data quality confirmed by generating quality control report using FastQC(Galaxy Version 0.74). For Quality and adapter trimming Trim Galore (Galaxy version 0.6.7) was used. The command ensures data quality in MACS2 by limiting duplicate reads to one per location (--keep-dup '1'), using control samples (-c) to model background noise, and setting a stringent q-value threshold (--qvalue '0.05') for significant peak detection. It also specifies parameters for accurate fragment size modeling (--mfold '5' '50') and minimum fragment length (--d-min 20), and generates BedGraph outputs (--bdg) for visualization.<br><br>ChIPseeker (Galaxy Version 1.28.3) was used for ChIP peak annotation and visualization. computeMatrix (Galaxy Version 3.5.4) was used to prepares data for plotting a heatmap or a profile of given regions. Heatmap (Galaxy Version 2.2.2) was used to plot the heatmap of the dataMatrix. MEME-ChIP (version 5.5.7) was used for motif analysis of large nucleotide datasets.<br><br>Visualization and comparing bed files and expression data were performed in R as it is described in the text and codes are available on https://doi.org/10.5281/zenodo.14030238

For manuscripts utilizing custom algorithms or software that are central to the research but not yet described in published literature, software must be made available to editors and reviewers. We strongly encourage code deposition in a community repository (e.g. GitHub). See the Nature Portfolio guidelines for submitting code & software for further information.

Data

Policy information about availability of data

All manuscripts must include a data availability statement. This statement should provide the following information, where applicable:

- Accession codes, unique identifiers, or web links for publicly available datasets
- A description of any restrictions on data availability
- For clinical datasets or third party data, please ensure that the statement adheres to our policy

All RNA-Seq datasets reported in this study, and H3K4me3 and H3K27me3 ChIP-Seq data have been deposited in the GEO repository under accession number GSE237560 [https://www.ncbi.nlm.nih.gov/geo/query/acc.cgi?acc=GSE237560]. The transcriptional signatures of activated B cells, PBs, and PCs have been defined using the corresponding (and naïve B cells for baseline comparison) RNA-Seq datasets from (Minnich, M. et al, GSE71698, [https://www.ncbi.nlm.nih.gov/geo/query/acc.cgi]). RIF1 ChIP-Seq was previously reported (Malzl, D. et al., GSE228880, [https://www.ncbi.nlm.nih.gov/geo/query/acc.cgi]). The BLIMP1-bound regions in activated B cells were defined based on the BLIMP1 Bio-ID dataset from (Minnich, M. et al, GSE71698). The lists of BLIMP1-activated and -repressed targets have been previously described Minnich, M. et al. Source data are provided with this paper.

Research involving human participants, their data, or biological material

Policy information about studies with human participants or human data. See also policy information about sex, gender (identity/presentation), and sexual orientation and race, ethnicity and racism.

Reporting on sex and gender	Human participants were not involved in this study
Reporting on race, ethnicity, or other socially relevant groupings	Human participants were not involved in this study
Population characteristics	Human participants were not involved in this study
Recruitment	Human participants were not involved in this study
Ethics oversight	Human participants were not involved in this study

Note that full information on the approval of the study protocol must also be provided in the manuscript.

Field-specific reporting

Please select the one below that is the best fit for your research. If you are not sure, read the appropriate sections before making your selection.

[x] Life sciences [ ] Behavioural & social sciences [ ] Ecological, evolutionary & environmental sciences

For a reference copy of the document with all sections, see nature.com/documents/nr-reporting-summary-flat.pdf

Life sciences study design

All studies must disclose on these points even when the disclosure is negative.

Sample size	Sample size was determined based on previous studies in the field
Data exclusions	No data exclusion from analyses

Replication	All experiments were conducted at least two independent times. All attempts at replication were successful.
Randomization	Randomization is not relevant for this study. For mice, age-matched controls were used for the experiments.
Blinding	Blinding is not performed in this study. Validation of genotype and identification of controls versus experimental samples is performed at the beginning of each experiment.

Reporting for specific materials, systems and methods

We require information from authors about some types of materials, experimental systems and methods used in many studies. Here, indicate whether each material, system or method listed is relevant to your study. If you are not sure if a list item applies to your research, read the appropriate section before selecting a response.

Materials & experimental systems

n/a	Involved in the study	
☐	☒	Antibodies
☐	☒	Eukaryotic cell lines
☒	☐	Palaeontology and archaeology
☐	☒	Animals and other organisms
☒	☐	Clinical data
☒	☐	Dual use research of concern
☒	☐	Plants

Methods

n/a	Involved in the study	
☐	☒	ChIP-seq
☐	☒	Flow cytometry
☒	☐	MRI-based neuroimaging

Antibodies

Antibodies used

Flow Cytometry Antibodies:
CD138-PE BioLegend 142503 281-2 (1:200)
CD138-BV421 BioLegend 142508 281-2 (1:200)
IgG2b-PE BioLegend 406708 RMG2b-1 (1:400)
IgG3-biotin BD Pharmigen 553401 R40-82 (1:400)
IgM-PeCy7 eBioscience 25-5790-82 II/41 (1:200)
streptavidin-BV605 BioLegend 405229 streptavidin (1:600)
streptavidin-APC BioLegend 405207 streptavidin (1:600)
streptavidin-BV605 BioLegend 405229 streptavidin (1:600)
IgG1-APC BD Pharmigen 560089 A85-1 (1:200)
IgG1-BV510 BD Biosciences 740121 A85-1 (1:200)
B220-BV785 BioLegend 103245 RA3-6B2 (1:200)
CD19-BV605 BioLegend 115539 6D5 (1:200)
CD19-BV650 BioLegend 115541 6D5 (1:200)
CD38-AF700 eBioscience 56-0381-82 90 (1:200)
FAS-PeCy7 BD Pharmigen 557653 Jo2 (1:200)
CXCR4-biotin BD Pharmigen 551968 (1:200)
CD86-BV421 BioLegend 105032 GL-1 (1:200)
IgA-PE eBioscience 12-4204-82 mA-6E1 (1:200)
TACI-Alexa647 BD Pharmigen 558453 8F10 (1:200)
AA4.1-PeCy7 BioLegend 136506 AA4.1 (1:200)
MHCII-BV510 BioLegend 107635 M5/114.15.2 (1:200)

Elispot Antibodies:
goat anti-mouse IgM-biotin Southern Biotech 1020-08 (1:500)
goat anti-mouse IgG1-biotin Southern Biotech 1071-08 (1:500)
streptaviding-AP Conjugate Roche 11089161001 (1:3000)

Chip-seq and Chip-qpcr antibodies:
a-HA Santa Cruz sc-7392 F-7 F0120, 0.5 µg anti-HA antibody per 25µgs of DNA
Anti-H3K4me3 antibody abcam ab8580, 10µg of antibody per 10 million cells
Anti-H3K27me3 antibody Cell Signaling, C36B11, 10µg of antibody per 10 million cells

Western blot antibodies:
a-RIF1 (1:10000), inhouse production Covance – Antibody Services, Princeton
a-β-actin (1:100000) Sigma Aldrich A5441
a-BLIMP1 (1:1000) Novus NB600-235
secondary goat anti-mouse IgG (1:1000) Jackson 115-035-174
secondary goat anti-rabbit IgG (1:1000) Jackson 111-035-008

Validation	All commercially available antibodies have been previously validated and references can be found on the vendors' websites<br>ELISPOT antibodies have been previously validated in : Winkler, W. et al. (2023) 'Mouse models of human multiple myeloma subgroups', Proceedings of the National Academy of Sciences, 120(10), p. e2219439120. Available at: https://doi.org/10.1073/pnas.2219439120.<br>a-RIF1 western blot antibody was previously validated in: Michela Di Virgilio et al. ,Rif1 Prevents Resection of DNA Breaks and Promotes Immunoglobulin Class Switching.Science339,711-715(2013).DOI:10.1126/science.1230624

Eukaryotic cell lines

Policy information about cell lines and Sex and Gender in Research

Cell line source(s)	40LB feeder cells (Balb/c 3T3 cell line expressing exogenous CD40-ligand (CD40L) and B-cell activating factor (BAFF) obtained from the Dr. Daisuke Kitamura laboratory
Authentication	40LB feeder cells were generated and characterised in the original study by Nojima, T., Haniuda, K., Moutai, T. et al. In-vitro derived germinal centre B cells differentially generate memory B or plasma cells in vivo. Nat Commun 2, 465 (2011). https://doi.org/10.1038/ncomms1475. No additional authentication was done.
Mycoplasma contamination	Cells were confirmed as Mycoplasma-negative.
Commonly misidentified lines<br>(See ICLAC register)	No commonly misidentified cell lines were used in this study.

Animals and other research organisms

Policy information about studies involving animals; ARRIVE guidelines recommended for reporting animal research, and Sex and Gender in Research

Laboratory animals	Rif1FH/FH, Cd19Cre, Rif1F/FCd19Cre/+ , Shld1-/- and Aicda-/- mice were previously described and maintained on a C57BL/6 background. Rosa26dCas9-Suntag/+ mice were generated by breeding Rosa26-LSL-dCas9 mice, purchased from the Jacksons laboratory (RRID: MMRRC_043926-JAX), with BALB/c-Tg(CMV-cre)1Cgn/J mice. Mice were kept in a specific pathogen-free (SPF) barrier facility and were between 8-18 weeks of age.
Wild animals	No use of wild animals in this study.
Reporting on sex	Both female and male mice were used in this study at similar ratios.
Field-collected samples	The study does not involve field-collected samples.
Ethics oversight	All experiments were performed in compliance with the European Union (EU) directive 2010/63/EU, and in agreement with Landesamt für Gesundheit und Soziales directives (LAGeSo, Berlin, Germany).

Note that full information on the approval of the study protocol must also be provided in the manuscript.

Plants

Seed stocks	No plant specimens were involved in this study.
Novel plant genotypes	No plant specimens were involved in this study.
Authentication	No plant specimens were involved in this study.

ChIP-seq

Data deposition

☒ Confirm that both raw and final processed data have been deposited in a public database such as GEO.

☒ Confirm that you have deposited or provided access to graph files (e.g. BED files) for the called peaks.

Data access links<br>*May remain private before publication.*	The data is deposited in GEO under accession number GSE237560 (https://www.ncbi.nlm.nih.gov/geo/download/?acc=GSE237560&format=file)
Files in database submission	GSM7624757 IgG Control, ChIP, H3K4me3

	GSM7624758 IgG Control, ChIP, H3K27me3<br>GSM7624759 IgG1, ChIP, H3K4me3<br>GSM7624760 IgG1, ChIP, H3K27me3, R1<br>GSM7624762 IgG1, ChIP, H3K27me3, R2
Genome browser session<br>(e.g. UCSC)	https://genome.mdc-berlin.de/cgi-bin/hgTracks?<br>hgS_doOtherUser=submit&hgS_otherUserName=arahjou&hgS_otherUserSessionName=RIF1_paper

Methodology

Replicates

Two biological replicates were included for H3K27me3 ChIP-seq. H3K4me3 ChIP-seq contain one experimental and one control sample.

Sequencing depth

Name	Total number of reads	read_lenght
GSM7624757	32,313,159	50
GSM7624759	26,050,367	50
GSM7624758	app. 20x10e6	100
GSM7624759	app. 20x10e6	100
GSM7624760	app. 20x10e6	100
GSM7624762	app. 20x10e6	100

Antibodies

Anti-H3K4me3 antibody (abcam, ab8580)
Anti-H3K27me3 antibody (Cell Signalling, C36B11)

Peak calling parameters

Peak calling for re-analysis of published data:
Peak calling command line for RIF1:

```
export PYTHON_EGG_CACHE=`pwd` &&   (macs2 callpeak   -t '/corral4/main/objects/4/c/2/dataset_4c22d11b-2d4d-4f28-bffe-436af4cd9b0c.dat' '/corral4/main/objects/0/7/8/dataset_078b0a4a-7810-4ae2-9294-e62768098e65.dat'  --name SRR24055120 -c '/corral4/main/objects/b/5/b/dataset_b5b86ecb-26ec-469b-9c9a-a217bb46ae4e.dat' '/corral4/main/objects/1/9/8/dataset_198fa608-f853-4573-a18d-702405898d8f.dat'  --format BAM   --gsize '1870000000'           --keep-dup '1'  --d-min 20 --buffer-size 100000  --bdg  --qvalue '0.05'  --mfold '5' '50'  --bw '300'  2>&1 > macs2_stderr)   && ( count=`ls -1 SRR24055120* 2>/dev/null | wc -l`; if [ $count != 0 ]; then mkdir '/corral4/main/jobs/060/502/60502319/outputs/dataset_b8c6c91d-de6e-400b-9328-9424efa393ac_files' && cp -r SRR24055120* '/corral4/main/jobs/060/502/60502319/outputs/dataset_b8c6c91d-de6e-400b-9328-9424efa393ac_files' && python '/cvmfs/main.galaxyproject.org/shed_tools/toolshed.g2.bx.psu.edu/repos/iuc/macs2/86e2413cf3f8/macs2/dir2html.py' '/corral4/main/jobs/060/502/60502319/outputs/dataset_b8c6c91d-de6e-400b-9328-9424efa393ac_files' macs2_stderr > '/corral4/main/jobs/060/502/60502319/outputs/dataset_b8c6c91d-de6e-400b-9328-9424efa393ac.dat'; fi; ) && exit_code_for_galaxy=$? && cat macs2_stderr 2>&1 && (exit $exit_code_for_galaxy)
```

Peak calling command line for BLIMP1:

```
export PYTHON_EGG_CACHE=`pwd` &&   (macs2 callpeak   -t '/corral4/main/objects/b/b/2/dataset_bb2ac9a2-dd44-4c8b-8724-73e6b48bcf32.dat' '/corral4/main/objects/b/7/2/dataset_b722b0f4-69e3-4277-9b21-bd954b80a332.dat'  --name SRR2143373_bam    --format BAM   --gsize '1870000000'           --keep-dup '1'  --d-min 20 --buffer-size 100000  --bdg  --qvalue '0.05' --mfold '5' '50'  --bw '300'  2>&1 > macs2_stderr)   && ( count=`ls -1 SRR2143373_bam* 2>/dev/null | wc -l`; if [ $count != 0 ]; then mkdir '/corral4/main/jobs/051/043/51043177/working/dataset_dc8c3206-287b-45c8-bb91-e7bc11b4d257_files' && cp -r SRR2143373_bam* '/corral4/main/jobs/051/043/51043177/working/dataset_dc8c3206-287b-45c8-bb91-e7bc11b4d257_files' && python '/cvmfs/main.galaxyproject.org/shed_tools/toolshed.g2.bx.psu.edu/repos/iuc/macs2/640d3af5d833/macs2/dir2html.py' '/corral4/main/jobs/051/043/51043177/working/dataset_dc8c3206-287b-45c8-bb91-e7bc11b4d257_files' macs2_stderr > '/corral4/main/jobs/051/043/51043177/outputs/galaxy_dataset_dc8c3206-287b-45c8-bb91-e7bc11b4d257.dat'; fi; ) && exit_code_for_galaxy=$? && cat macs2_stderr 2>&1 && (exit $exit_code_for_galaxy)
```

Data quality

Sequencing data quality confirmed by generating quality control report using FastQC(Galaxy Version 0.74). For Quality and adapter trimming Trim Galore (Galaxy version 0.6.7) was used. The command ensures data quality in MACS2 by limiting duplicate reads to one per location (--keep-dup '1'), using control samples (-c) to model background noise, and setting a stringent q-value threshold (--qvalue '0.05') for significant peak detection. It also specifies parameters for accurate fragment size modeling (--mfold '5' '50') and minimum fragment length (--d-min 20), and generates BedGraph outputs (--bdg) for visualization.

Software

FastQC(Galaxy Version 0.74), Trim Galore: Quality and adapter trimmer of reads (Galaxy Version 0.6.7), Bowtie2: map reads against reference genome (Galaxy Version 2.5.3), MACS2 callpeak: Call peaks from alignment results (Galaxy Version 2.2.9.1)

Flow Cytometry

Plots

Confirm that:

- ☒ The axis labels state the marker and fluorochrome used (e.g. CD4-FITC).
- ☒ The axis scales are clearly visible. Include numbers along axes only for bottom left plot of group (a 'group' is an analysis of identical markers).
- ☒ All plots are contour plots with outliers or pseudocolor plots.
- ☒ A numerical value for number of cells or percentage (with statistics) is provided.

Methodology

Sample preparation	Cell suspensions were washed once with FACS buffer (PBS supplemented with 1% FBS and 1mM EDTA), blocked with TruStain fcX (BioLegend) for 10 min at 4° and stained with conjugated antibodies diluted in FACS buffer for 20min at 4°. Cell suspensions were washed twice with FACS buffer to remove unbound antibodies. 1 µg/ml of propidium iodide (PI) was used for live/dead cell staining when stated.
Instrument	BD LSRFortessa™ Cell Analyzer and BD FACSAria™ III Cell Sorter
Software	FlowJo™ v10.10
Cell population abundance	N/A
Gating strategy	Gating strategy is provided in all relevant figures either as main or supplementary panels.

☒ Tick this box to confirm that a figure exemplifying the gating strategy is provided in the Supplementary Information.
